# Tellurium

**Published:** 1887-01

**Authors:** Ad. Lippe

**Affiliations:** Philadelphia


### HOMŒOPATHIC PHYSICIAN,

A MONTHLY JOURNAL OF MEDICAL SCIENCE.

"If our school ever gives up the strict inductive method of Hahnemann, we are lost, and deserve only to be mentioned as a caricature in the history of medicine."—CONSTANTINE HERING.

Vol. VII. JANUARY, 1887. No. 1.

### TELLURIUM.

AD. LIPPE, M. D., PHILADELPHIA.

The mental symptoms may be comprised in "quietude," less inclined to be lively or to fly into a passion. This condition might properly come under the head of "*apathy*," which we find strongly under Arsenicum, Phosph.-acid, Ignatia, Natrum mur., etc.

There is vertigo when rising in the morning, aggravated from every movement, similar to Carbo veg. Glonoin has a similar vertigo with diabetes. There is fullness of the head with sleepiness, similar to Belladonna.

The most characteristic headache is a violent linear pain in a small spot over the left eye—the locality is similar to Kalmia and Lycopod. The Tellurium headache comes suddenly, and leaves as suddenly as it came.

The eye symptoms are very important and may indicate it in pterygeum and cataract. We have not as yet any clinical verification of the eye symptom. Among the ear symptoms we find a characteristic indication for it which has been verified repeatedly.* Itching and swelling, with painful throbbing in external meatus; in three or four days discharges of a watery fluid, smelling like fish-pickle, which causes vesicles wherever it touches; ear is bluish red as if œdematous; hearing impaired. Tellurium has not the stitches in the ear nor the early swelling and redness of the ear that we find under Pulsatilla. The discharge under Pulsatilla is like pus, green. Tellurium differs

* In Allen's *Encyclopædia*, this symptom is to be found under "Skin."

from Mercury, which has characteristic a great aggravation of the pains and sensation of coldness of the inflamed external meatus when becoming warm in bed.

The discharge from the ear is frequently blood-streaked. Bovista has also a very offensive discharge from the ear, but the soreness which it causes manifests itself in the formation of scabs.

We find uncommon abundant secretion of saliva from the mouth, like Mercury, and also a whitish coated and swollen tongue, so that the impressions of the teeth are clearly shown upon the margin of the tongue, like Mercury, which has a gray or heavy coated tongue with foul breath. The breath and other exhalations of Tellurium are like garlic. Swollen tongue, with indentations of the teeth we find, besides Tellur. and Mercury, also under Stramonium, Arsenicum metallicum, Glonoine, and Iodine.

Gums bleed so that the mouth is filled with blood: similar to Nat. mur., Carbo veg., and Mercury.

The sore throat of Tellurium is always relieved by eating and drinking; very similar to Ignatia. Tellurium has dryness of the throat. Ignatia has the sensation of a plug in the throat.

The breath smells like garlic; this is a characteristic symptom of Tellurium, and so far not known to have been produced by any other remedy.

Tellurium has weak feeling, like faintness, in the stomach, after congestion of blood in head and nape. This weak feeling in the stomach is also found under Sepia accompanied by nausea, and is then caused by thinking of food to be offered. Baryta has it also, but eating relieves it. Oleander has relief of it from drinking brandy. Alumina and Digitalis have an aggravation of it after eating. Kalmia has it extending to the throat relieved by eructations; Kali carb. has that sensation of weakness accompanied by eructations. Lobelia has it with dyspnœa.

Tellurium has painful sensitiveness of the spine from last cervical to fifth dorsal vertebra, sensitive to pressure and touch, similar to Nux vom. and Ruta.

A very important symptom is, "pain in the sacrum passing into the right thigh down the sciatic nerve; worse when pressing at stool, coughing, laughing, also when lying on the affected side." Here we have a true picture of a form of sciatica. Lachesis is also frequently indicated in sciatica, but the Lachesis pains in the sciatic nerve are relieved and are very slight when lying quiet in bed. Sitting up—rising to one's feet—causes a great aggravation. The pain, if aggravated, is a sensation of intense heat, as from a hot iron, and is often aggravated by sleep. Rhus tox. will be also a remedy in this painful disease if the pain is worse at night, when rising from bed or from a seat, and relieved by continued motion and walking. Arsenic will be the remedy if the person can find no rest in any position, but feels himself compelled to walk about, changing the position, which is painful and gives no relief. Lycopodium has cured sciatica, returning every four days periodically. Kali bichromicum has also pain in the sciatic nerve as far as the knee.

Tellurium has cured offensive perspiration of the armpit when the smell was described to be like garlic. Other offensive smelling perspirations of the armpit are found under Hepar, Dulcamara, Nitr. ac., Rhododendron, Selen., Sepia, and Thuja. Very characteristic of Tellurium is "ring-worm." The ring-worm consists of red, elevated rings very distinctly marked. It appears as spots, very bright red and sharply defined, with minute itching vesicles; itching is worse especially at night after going to bed. The ring-worm of Natrum carbonicum is surrounded by a yellow ring or it suppurates. Sepia has brown or claret colored spots, or humid ring-worm with itching. Clematis has red humid herpes and ring-worm with intolerable itching in the warmth of the bed and after washing; the herpes is red and humid with the increasing, but pale and dry with the decreasing moon. Magnesia carbonica has small red, little elevated, smooth herpes, scaling off afterward without sensation.

---

COMMENTS.—In the fifth volume of the *Homœopathic Review* will be found the original reports of the provers, and also the arrangement of symptoms by the late Dr. Constantine Hering. The profession at large should be grateful for the labors of such an indefatigable member of it. But we must regret to state that there comes from Gotham a censorship by a self-appointed progressive-recognition-seeking physician, at present Dean of the so-called Homœopathic College, who declares Hering's *Guiding Symptoms* unscientific and unreliable! This censor indorses Richard Hughes' materia-medica-of-the-future, while he evidently feels sore and laments with his publisher that Hering's *Guiding Symptoms* are pushed forward because it is well known that they are needed, and that every true healer will welcome the appearance of so useful a work. Unscientific, why? because it is useful and is not clad in the pathological livery? This *scientific* censor has but a short time ago espoused the exploded germ-theory—picking up the crumbs that fall from the allopathic table. Such a censor will not be able to retard the publication of Hering's *Guiding Symptoms*. Such men, late graduates, who by this ungrateful teacher were impressed with the idea that the *Organon* is a poor concern anyhow, old, out of fashion, and now properly laid aside for a "progressive science"—in short, of no great value. Late graduates, or, say, a majority of them, may be misled into the belief that Homœopathy may be inscribed on their diplomas, and a dispensation added to it to discard the *Organon* and the published works of the men who gave our school its standing, by following Hahnemann's methods faithfully and developing our healing art, by adding to the materia medica such provings as will enable us to be still more successful than ever before. There are some graduates, even of such colleges, who have more intellect and a better understanding of "science" than their misguided and misguiding teachers. They will not accept the proffered and recommended progressive science, including the germ-theory and the Quinine departure. They will accept as homœopathicians the *Organon*, Hahnemann's *Materia Medica*, and Hering's *Guiding Symptoms*, and become healers indeed. They will learn that their successes in healing the sick increase as their knowledge of Hahnemann's methods and of his *Materia Medica* increases. *They* will give credit and remember gratefully such early promulgators of Homœopathy as Hahnemann taught, as the late Dr. Constantine Hering, and atone for the ungrateful remarks made by their former teacher and now censor of Hahnemann and Hering.

